# PIN (Protein Inhibitor of Neuronal Nitric Oxide Synthase) Modulates Glucose Uptake Through NO-Dependent and Independent Mechanisms in Rat Muscle Cells

**DOI:** 10.3390/antiox15040436

**Published:** 2026-03-31

**Authors:** Jérémy Leroy, Karima Mezghenna, Didier Tousch, Jaufret Canovas, Daniel Laune, Martine Pugnière, Jacqueline Azay-Milhau, Anne-Dominique Lajoix

**Affiliations:** 1 Biocommunication in Cardio-Metabolism (BC2M), University of Montpellier, 34093 Montpellier, France; jeremy.leroy@umontpellier.fr (J.L.); karima.mezghenna@umontpellier.fr (K.M.);; 2 Qualisud, University of Montpellier, CIRAD, Montpellier SupAgro, 34093 Montpellier, France; didier.tousch@umontpellier.fr; 3 Kyomed, 34184 Grabels, France; 4 IRCM, Institut de Recherche en Cancérologie de Montpellier, INSERM, U1194, University of Montpellier, 34093 Montpellier, France; 5 ICM, Institut Régional du Cancer, 34093 Montpellier, France

**Keywords:** protein inhibitor of neuronal NO synthase, glucose uptake, skeletal muscle, nitric oxide synthase, myosin Va

## Abstract

Protein inhibitor of neuronal NO synthase (PIN) or dynein light chain 8 (LC8) is a highly conserved protein interacting with multiple partners, like neuronal NO synthase (nNOS) or myosin Va to modulate a variety of cellular functions. As PIN is expressed in skeletal muscle, our aim was to investigate a possible role of PIN in glucose uptake in L6 and primary muscle cells. PIN overexpression resulted into a decrease in glucose uptake with reduced GLUT4 expression and translocation at the plasma membrane, similarly to the pharmacological blockade of nNOS with L-NAME. PIN effect is mediated by a reduction in nNOS protein level and a direct interaction with nNOS leading to a reduced NO production in L6 myocytes. Surprisingly, a siRNA targeting PIN decreased glucose uptake and GLUT4 translocation, suggesting the involvement of nNOS-independent effects. We therefore focused on myosin Va which interacts with PIN in L6 myocytes. Myosin Va silencing provoked a decrease in glucose uptake. As PIN siRNA also reduced myosin Va expression, this confirms the essential role of myosin Va in the observed effects of PIN silencing on glucose uptake. We conclude that PIN modulates glucose uptake and GLUT4 translocation in rat muscle cells, through NO-dependent and -independent mechanisms.

## 1. Introduction

PIN (Protein Inhibitor of Neuronal NO synthase) or LC8 (Dynein Light Chain 8) is a 89-amino acid protein, highly conserved between *Chlamydomonas*, nematode, *Drosophila* and mammalians. The protein was found in rat brain as an interacting partner of neuronal NO synthase (nNOS), which prevents the enzyme homodimerization and subsequently inhibits NO production [1]. Furthermore, PIN has also been identified as the light chain of cytoplasmic and flagellar dyneins (called LC8, DLC1, or DYNLL1) as well as of myosin Va, two cytoskeletal motors involved in intracellular trafficking [2]. In addition, PIN interacts with a variety of partners, including the proapoptotic factor Bim [3], adaptor proteins (e.g., DAP1α and Swallow) [4,5], transcriptional factors (e.g., NRF-1 and CITFA) [6,7], and viral proteins [8]. As consequence, PIN is involved in a variety of cellular functions such as modulation of activity and/or localization of proteins [1,3,5,8], transcriptional regulation [6,7], intracellular trafficking of cargoes [9,10], or cancer development [11]. Moreover, partial loss of function of PIN in *Drosophila* leads to global morphogenic defects, female sterility and disorganization in actin cytoskeleton, whereas total loss of function is lethal in flies [12] and also in mice (EUCOMM data), confirming its essential role in cell development and physiology.

Both nNOS and PIN are expressed in rodent and human skeletal muscles and are mainly, but not exclusively, distributed along the sarcolemma of positive fibers [13,14]. On a metabolic level, skeletal muscles are involved in glucose homeostasis as the major site of insulin-stimulated glucose disposal in the body (75–80%) [15]. Insulin is the main triggering signal for glucose transport, by inducing the translocation of glucose transporter GLUT4 from intracellular stores to the plasma membrane. In the basal state, 90% of GLUT4 is sequestered in perinuclear storage vesicles and extracellular signals lead to their fusion to the plasma membrane and exocytosis [16]. nNOS has been reported to be involved in the regulation of contractile force, glucose transport, energy metabolism, and blood flow [17]. However, the functional role of PIN in skeletal muscle is still unclear. PIN remains highly expressed in muscles from patients with Duchenne muscular dystrophy, which markedly contrasts with the absence of nNOS [13], suggesting some independent roles for the two proteins.

In the present study, we examined the role of PIN on glucose uptake and GLUT4 translocation in primary rat myocytes and the L6 rat myocyte cell line that closely mimic the characteristics of mature skeletal muscle fibers [18]. Then, we investigated whether PIN functional effects in skeletal muscle could be mediated by its interaction with nNOS and modulation of nNOS activity on glucose uptake. Interestingly, we found that PIN exerts NO-dependent and -independent activities on glucose uptake, related to its interaction with another binding partner, myosin Va.

## 2. Materials and Methods

### 2.1. Culture of L6 Cells

L6 myoblasts (ATCC, CRL-1458) were cultured in DMEM containing 25 mM glucose supplemented with 10% fetal calf serum, 100 units/mL penicillin, 100 µg/mL streptomycin, and 2 mM glutamine at 37 °C in a 5% CO_2_ atmosphere according to ATCC’s recommendations. They were cultivated during 4 days before differentiation and for 4 additional days in 2% FCS-complete medium (except for immunofluorescence experiments that lasted a total of 14 days). This timing allowed differentiation of the cells into pre-fusion myocytes and myotubes and is consistent with transfection experiments to efficiently modulate PIN expression.

### 2.2. Animals

Male Wistar rats, male Zucker *fa/fa* rats, male ZDF *fa/fa* rats and ZDF *fa/+* lean controls (9 weeks old) were purchased from Charles River (L’Arbresle, France). Animals were housed (3–4 per cage) in our conventional animal facility (n°D34-172-25) under conditions of constant temperature (20–22 °C), humidity (45–50%), and a standard dark cycle (20.00–08.00 h) with free access to food and water. Wistar and Zucker *fa/fa* rats were fed regular foods (SAFE A04, Safe, Rosenberg, Germany) whereas ZDF *fa/fa* rats received Purina 5008 food to induce the consistent development of diabetes (LabDiet, London, UK). The experiments were performed in accordance with the “Guide for the Care and Use of Laboratory Animals” (8th edition, 2011). The protocol was approved by the ethical committee of our University (APAFIS#-22338- 01910081144807 v2 approved 29 April 2020). After euthanasia of the rats, gastrocnemius muscles (for isolation of satellite cells) or brain (for purification of myosin Va) were immediately collected by standard dissection.

### 2.3. Isolation and Culture of Satellite Cells

Satellite cells were isolated from rat gastrocnemius muscles which were removed, cleaned, minced, and digested for 1 h at 37 °C with 1.25 mg/mL protease from Streptomyces griseus (Sigma-Aldrich, Steinheim, Germany) [14]. Isolated cells were centrifuged at 1500× *g* for 4 min and resuspended in PBS. Satellite cells were separated by three cycles of centrifugation at 500× *g* for 10 min (satellites were in the supernatant). After a final centrifugation at 1500× *g*, they were then plated on an uncoated dish in DMEM containing 10% horse serum for 6 h. Unattached floating cells were then cultured in Ham’s F-10 nutrient mixture containing 20% fetal calf serum (growth medium) on ECM gel-coated dish (Sigma-Aldrich). After 5 days, cells were differentiated with DMEM containing 2% horse serum for another 5-day period to yield pre-fusion myocytes and myotubes.

### 2.4. Treatments of Muscle Cells

At the beginning of differentiation, PIN was overexpressed by using an expression vector containing PIN cDNA (pcDNA3.1, Invitrogen, Carlsbad, CA, USA), introduced into L6 cells by Lipofectamine 3000/P3000 Reagent (Invitrogen). PIN and myosin Va expression was also silenced in L6 myocytes by specific siRNA (PIN siRNA, Ambion, Austin, TX, USA; myosin Va siRNA, Qiagen, Hilden, Germany) using RNAiMAX reagent (Invitrogen) during respectively 72 and 48 h. L6 cells were also treated overnight with 20 µM of cell permeant inhibitory Tat-peptide, GRKKRRQRRRGGIDVGIQVDWD or an irrelevant Tat-peptide, used as a control (EZBiolabs, Carmel, IN, USA).

### 2.5. Western Blotting

L6 cells were homogenized in 20 mM Tris lysis buffer pH 7.4, containing 150 mM NaCl, 1% Triton X-100, 0.1% SDS and a cocktail of protease inhibitors.

Proteins (15 to 40 µg) were separated on a 7.5% SDS-polyacrylamide gel for nNOS/myosin Va and on a 14% tricine polyacrylamide gel for PIN. Incubation with anti-nNOS (BD Biosciences, Franklin Lakes, NJ, USA), anti-PIN (Epitomics, Burlingame, CA, USA), anti-GLUT4 (Interchim, Montluçon, France), anti-GLUT1 (Abcam, Cambridge, UK), or anti-myosin V (Cell Signaling Technology, Beverly, MA, USA), or anti-α-tubulin (Sigma-Aldrich, as an internal control) antibodies was performed overnight. Signals were acquired with ChemiDoc Go Imaging System (Bio-Rad, Marnes-la-Coquette, France) and quantified by Image Lab (Bio-Rad). Western blot presented in the figures are representative of 3 independent experiments. Associated histograms represented quantification.

For immunoprecipitation experiments, L6 cells were homogenized in lysis buffer, containing 1% Triton X-100, 0.5% Nonidet P-40, and protease inhibitors. nNOS or myosin V was immunoprecipitated from 2 mg protein extract with a polyclonal anti-nNOS (BD Biosciences) or anti-myosin V (Cell Signaling) antibody overnight and proteins were immunoblotted as described above.

### 2.6. Immunofluorescence

L6 cells were seeded on poly-L-lysine (Sigma-Aldrich) coated Lab-Tek™ System. They were immunostained overnight with anti-nNOS (Immunostar, Hudson, WI, USA), anti-PIN (Santa Cruz Biotechnologies, Santa Cruz, CA, USA) or anti-myosinV (Sigma-Aldrich) antibodies. The negative control was performed with only the secondary antibodies. Fluorescence was observed with the Zeiss LSM780 confocal microscope (Montpellier Rio Imaging) using a 63× oil immersion objective (Zeiss) and analyzed with Image J software, 1.54p version (Syngene, Cambridge, MA, USA). Colocalization was estimated using the JACoP plugin [19] and Costes’ randomization method [20] to calculate the Pearson coefficient. The Pearson coefficient indicates how close to the colocalization line is the intensity distribution dot cloud (close to 1 = colocalization).

### 2.7. Glucose Uptake

Glucose uptake experiments were performed in 12-well plates. L6 myocytes were starved during 4 h in DMEM supplemented with 0.1% BSA and then incubated during one hour in Krebs Ringer Bicarbonate (KRB) buffer containing 0.1% BSA, with or without 100 nM insulin and the compounds to be tested: D-ω-nitro-L-arginine methyl ester (D-NAME) (5 and 10 mM), N-ω-nitro-L-arginine methyl ester (L-NAME) (5 and 10 mM), sodium nitroprusside (SNP) (1–6 mM) (Sigma-Aldrich). Cells were gently washed and then incubated in 1 mL KRB buffer alone, containing 0.5 μCi [^3^H] deoxyglucose per well. Uptake was stopped by three washings in cold PBS and cells were lysed in 0.1N NaOH. Radioactivity was measured and normalized to total protein concentrations. Non-specific uptake was estimated using 10 µM cytochalasine B (Sigma-Aldrich), an inhibitor of actin polymerization, and subsequently deduced from all radioactive counts, as previously described [21].

### 2.8. Translocation of GLUT4

GLUT4 translocation was measured using a colorimetric immunoassay according to the protocol we previously described [14]. L6 cells were preincubated and stimulated with 100 nM insulin during 30 min, as described in Section 2.7. Then, cells were lightly fixed with absolute ethanol during 3 min to avoid permeabilization of intracellular GLUT4 vesicles. After saturation with PBS-0.4% BSA during 30 min, cells were incubated with an anti-GLUT4 antibody directed against 13 N-terminus extracellular amino acid of GLUT4 (GT42A, Alpha Diagnostic, San Antonio, TX, USA) overnight. After final incubation with peroxydase-conjugated anti-rabbit antibody (Sigma-Aldrich), the reaction was revealed by ortho-phenylenediamine with reading at 492 nm. The protocol was validated using a GLUT4 antibody raised against intracellular C-terminal epitope (1F8, Santa Cruz).

### 2.9. NOS Catalytic Activity Assay

**Figure 3:**
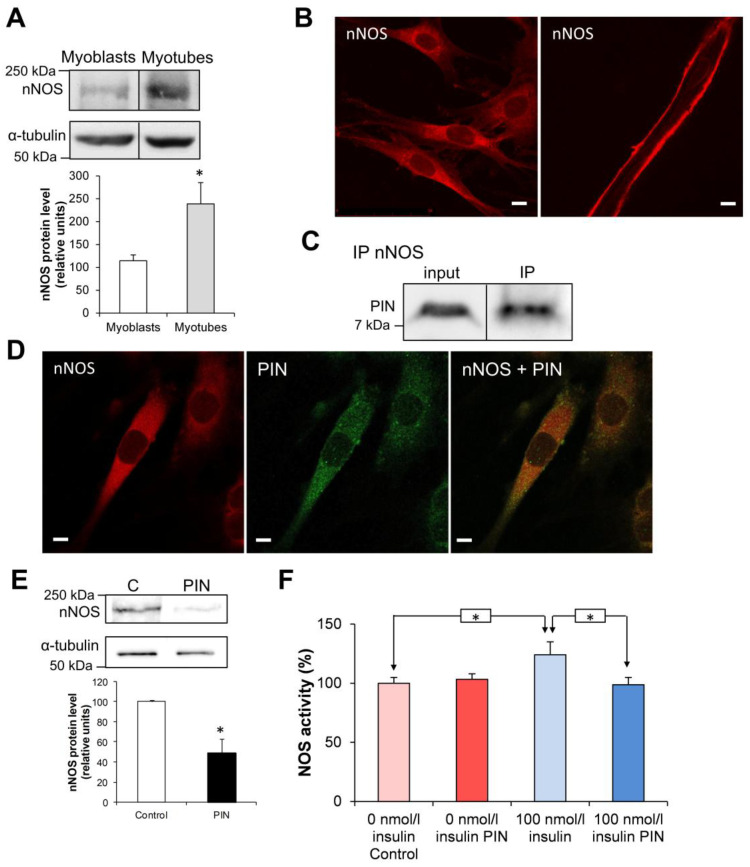
Involvement of nNOS in the effects of PIN overexpression in L6 muscle cells. (**A**) Western blotting analysis of nNOS expression in L6 myoblasts and L6 myotubes. (**B**) Cellular localization of nNOS in L6 myocytes (left picture) and L6 myotubes (right picture) by immunofluorescence. Scale bar is for 10 µm. (**C**) Coimmunoprecipitation (IP) of nNOS with PIN in L6 myocytes. (**D**) Colocalization of nNOS (red) and PIN (green) in L6 myocytes by immunofluorescence. (**E**) Western blotting analysis of nNOS expression in L6 myocytes after transfection with PIN cDNA (PIN) or the empty vector (C). (**F**) Effect of PIN overexpression on nNOS catalytic activity in L6 myocytes in absence and presence of 100 nM insulin. * *p* < 0.05.

**Figure 4:**
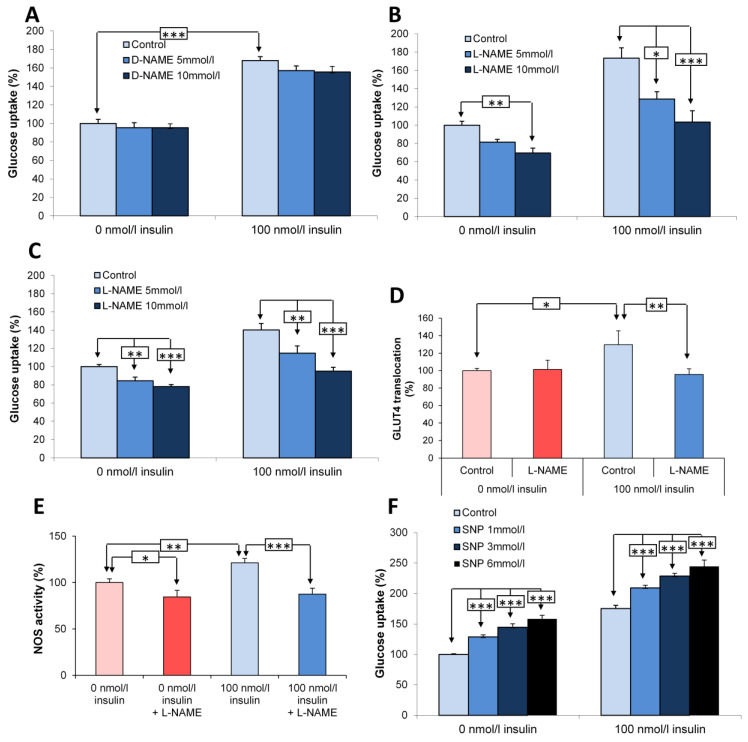
Effect of nNOS blockade by L-NAME on glucose uptake and GLUT4 translocation in L6 and primary muscle cells. Effects of D-NAME (**A**) and L-NAME (**B**) on glucose uptake in L6 myocytes with or without 100 nM insulin. (**C**) Effects of L-NAME on glucose uptake in primary myotubes with or without 100 nM insulin. (**D**) Colorimetric immunoassay measuring GLUT4 translocation in L6 myocytes in the presence of 10 mM L-NAME with or without 100 nM insulin. (**E**) Effect of L-NAME on nNOS catalytic activity with or without 100 nM insulin. (**F**) Effects of SNP on glucose uptake in L6 myocytes with or without 100 nM insulin. * *p* < 0.05; ** *p* < 0.01; *** *p* < 0.001.

**Figure 5:**
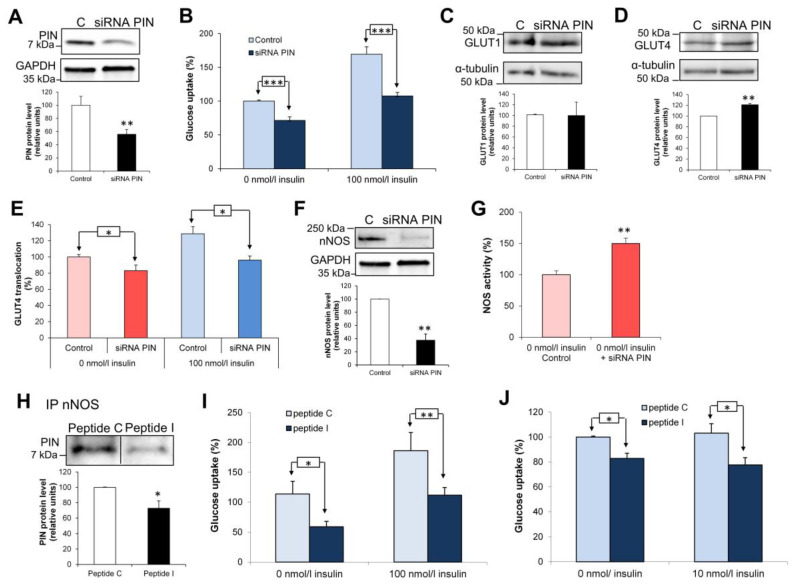
Effects of PIN silencing and disruption with its interacting partners on glucose uptake in L6 muscle cells. (**A**) Western blotting analysis of PIN under-expression after transfection with PIN and control (C) siRNA. (**B**) Effects of PIN silencing on glucose uptake in L6 myocytes with or without 100 nM insulin. (**C**) Western blotting analysis of GLUT1 expression after transfection with PIN and control (C) siRNA. (**D**) Western blotting analysis of GLUT4 expression after transfection with PIN and control (C) siRNA. (**E**) Colorimetric immunoassay measuring GLUT4 translocation in L6 myocytes after PIN silencing in the presence or not of 100 nM insulin. (**F**) Western blotting analysis of nNOS expression in L6 myocytes after transfection with PIN or control (C) siRNA. (**G**) Effect of PIN silencing on nNOS catalytic activity in the absence of insulin. (**H**) Coimmunoprecipitation of nNOS with PIN in the presence of the control (peptide C) versus the inhibitory peptide (peptide I) in L6 myocytes. (**I**) Effects of the inhibitory peptide (peptide I) on glucose uptake in L6 myocytes with or without 100 nM insulin versus the irrelevant one (peptide C). (**J**) Effects of the inhibitory peptide (peptide I) on glucose uptake in primary myocytes from the insulin resistant Zucker *fa/fa* rat. * *p* < 0.05; ** *p* < 0.01; *** *p* < 0.001.

nNOS catalytic activity was estimated in lysate from L6 myocytes by measuring the production of radiolabeled [^3^H]citrulline from [^3^H]arginine (MP Biomedicals, Illkirch-Graffenstaden, France) during one hour, according to the manufacturer’s recommandation (Cayman, Ann Harbor, MI, USA) (Figure 3 and Figure 4). For Figure 5, nNOS catalytic activity was measured in cell lysates using a commercial assay kit (Abcam, ab211084), according to the manufacturer’s instructions. Briefly, lysates were incubated with the reaction mix containing L-arginine and required cofactors to allow enzymatic conversion to NO and L-citrulline. nNOS activity (µU/µg) was calculated from the amount of nitric oxide produced (pmol), divided by the reaction time (60 min). All data were normalized to the total protein content (µg) in the lysate.

### 2.10. Expression and Purification of GST-PIN

Rat PIN cDNA (identical to human PIN cDNA) was obtained from INS-1 cells using RT-PCR and cloned in a pGEX2T vector (GE Healthcare, Velizy-Villacoublay, France) between BamHI and EcoRI sites, in frame with the N-terminus GST tag. Recombinant plasmid was transformed into BL21(DE3) cells (Novagen, Madison, WI, USA) and expressed during 4 h at 30 °C. GST-PIN was then purified by a glutathione sepharose column (GE Healthcare) and possibly digested by thrombin (GE Healthcare) to remove GST.

### 2.11. ELISA

For these experiments, we used recombinant rat nNOS (Enzo Life Sciences, Villeurbanne, France) or myosin V we purified from rat brain as previously described [22]. nNOS at a concentration of 1 µg/mL or myosin V at a concentration of 5 µg/mL were adsorbed into a Maxisorp microplate (Nunc) overnight and then incubated with 0.5 µg/mL GST-PIN and increasing concentrations of peptides (0.01 to 1000 µg/mL). The complex was revealed by a peroxydase-conjugated anti-GST antibody and a colorimetric reaction.

### 2.12. Surface Plasmon Resonance Analysis

nNOS-PIN interaction was analyzed by surface plasmon resonance (SPR) using BIACORE 2000 (GE Healthcare) at a flow rate of 30 µL/min. Recombinant rat nNOS (Enzo Life Sciences), at a concentration of 10 µg/mL was covalently immobilized (6000–6500 RU level) on a flowcell of a CM5 sensor chip (GE Healthcare) using the NHS-EDC protocol. A second flowcell without immobilized protein was used as a control. Recombinant PIN (5 µg/mL) was injected in the presence of increasing concentrations of peptides (1 to 100 µg/mL). Sensorgrams corresponded to a 180 sec association and a 400 sec dissociation step. Each experiment was repeated at least three times. The kinetic parameters of the binding reaction were determined using BIAevaluation 3.2 software (Biacore AB).

### 2.13. Statistical Analysis

Western blot and immunofluorescence experiments were performed in triplicate or quadruplicate. Quantitative data correspond to the mean values obtained from n independent experiments and are presented as mean ± SEM. Statistical comparisons between two groups were carried out using a two-tailed Student’s t-test, whereas multiple-group comparisons were evaluated by one-way or two-way ANOVA when appropriate, followed by the relevant post hoc tests. A *p* value < 0.05 was considered statistically significant. All analyses were performed using GraphPad software, 5.01 version.

## 3. Results

### 3.1. Expression and Localization of PIN During Differentiation of L6 Muscle Cells

**Figure 1:**
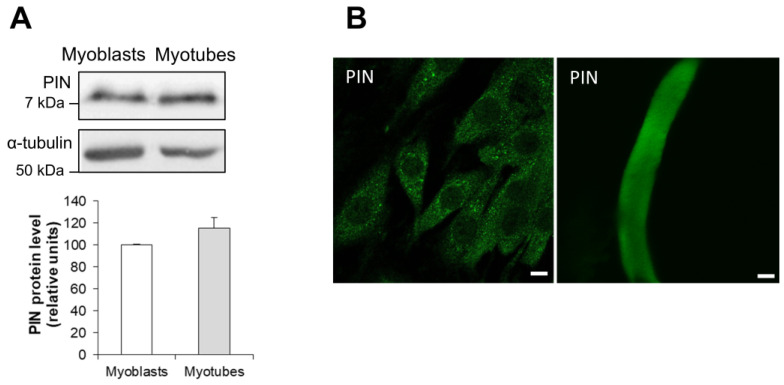
Analysis of PIN expression and cellular localization in L6 muscle cells. (**A**) Western blotting analysis of PIN expression in L6 myoblasts and L6 myotubes. (**B**) Cellular localization of PIN in L6 myocytes (left picture) and L6 myotubes (right picture) by immunofluorescence. Scale bar is for 10 µm.

L6 myoblats were differentiated into myotubes and changes in PIN protein expression were assessed by Western blotting. The PIN protein remained unchanged during differentiation of L6 cells (Figure 1A). By immunofluorescence, we observed that PIN staining appears punctuated and present throughout the cytoplasm, whereas in L6 mature myotubes, a less marked peripheral localization could be observed (Figure 1B).

### 3.2. Effect of PIN on Glucose Uptake and GLUT4 Translocation in L6 and Primary Muscles Cells

**Figure 2:**
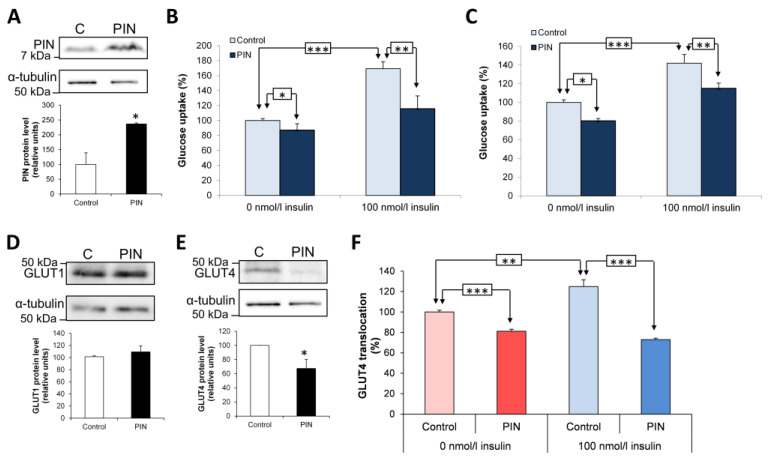
Effect of PIN overexpression on glucose uptake and GLUT4 translocation in L6 and primary muscle cells. (**A**) Western blotting analysis of PIN overexpression after transfection with PIN cDNA (PIN) or the empty vector (C) in L6 myocytes. (**B**) Effects of PIN overexpression on glucose uptake in L6 myocytes with or without 100 nM insulin. (**C**) Effects of PIN overexpression on glucose uptake in primary myotubes with or without 100 nM insulin. (**D**) Western blotting analysis of GLUT1 expression after transfection with PIN cDNA (PIN) or the empty vector (C) in L6 myocytes. (**E**) Western blotting analysis of GLUT4 expression after transfection with PIN cDNA (PIN) or the empty vector (C) in L6 myocytes. (**F**) Colorimetric immunoassay measuring GLUT4 translocation after PIN overexpression in L6 myocytes with or without 100 nM insulin. * *p* < 0.05; ** *p* < 0.01; and *** *p* < 0.001.

To investigate the role of PIN in glucose uptake, L6 cells were transfected with an expression vector encoding PIN, leading to a 2.5-fold increase in PIN expression (*p* < 0.05; *n* = 3) (Figure 2A). In this cellular model, insulin (100 nM) stimulated glucose uptake by 70 ± 16% (*p* < 0.001; *n* = 5; Figure 2B). Glucose uptake was decreased after PIN overexpression by 13 ± 4% (*p* < 0.05) and 54 ± 6% (*p* < 0.01) (*n* = 6) in the absence of and in the presence of 100 nM insulin, respectively (Figure 2B). We also used primary rat myocytes obtained from the culture, and differentiation of satellite cells isolated from skeletal muscles of Wistar rats [14]. In primary myocytes, PIN overexpression similarly reduced glucose uptake by 20 ± 2.5% (*p* < 0.05) in the absence of and by 27 ± 6% (*p* < 0.01) (*n* = 5) in the presence of insulin (Figure 2C).

In addition to glucose uptake, we investigated whether PIN overexpression could modify expression of the glucose transporter GLUT1, mainly localized at the plasma membrane and accounting for 30–40% of basal glucose uptake [23]. GLUT1 expression was found unchanged when PIN was overexpressed in L6 cells (Figure 2D). In contrast, the level of GLUT4, which is more intracellularly localized in basal state, was diminished by 33% in the presence of PIN overexpression (*p* < 0.05) (Figure 2E).

We finally analyzed whether PIN overexpression could modify GLUT4 translocation at the membrane. In the presence of PIN overexpression, GLUT4 translocation was reduced in the absence (−19 ± 2%) and in the presence of insulin (−52 ± 2%) (*p* < 0.001; *n* = 4) (Figure 2F).

We can thus conclude that PIN displays a negative effect on glucose uptake by decreasing GLUT4 expression and translocation in L6 and primary muscle cells.

### 3.3. Involvement of nNOS in the Effects of PIN Overexpression in L6 Muscle Cells

As PIN is the protein inhibitor of nNOS, we then investigated whether this partner of PIN could be involved in its negative role on glucose uptake. Indeed, nNOS has been previously shown to be involved in the regulation of glucose transport at the level of skeletal muscle [15,24]. During differentiation of L6 cells, the faint protein level of nNOS detected in myoblasts strongly increased in cells differentiated into myotubes (Figure 3A; *p* < 0.05). nNOS protein displayed a perinuclear punctuated distribution in L6 myocytes and appeared spread to the periphery of myotubes (Figure 3B), as we previously observed in muscle sections from Zucker rats [14].

As PIN binds with nNOS dimers to modulate its catalytic activity [1], we confirmed the direct interaction of PIN and nNOS in L6 myocytes by a coimmunoprecipitation experiment performed on a cell extract (Figure 3C). By immunofluorescence, double staining of nNOS and PIN in L6 myocytes revealed a colocalization of the two proteins. Analysis of the double staining pictures using Image J colocalization JACoP plugin revealed a Pearson coefficient of 0.8 ± 0.03 (*n* = 3) (Figure 3D), as previously shown by others [13]. When PIN was overexpressed in L6 cells, we observed a decrease in nNOS protein level (*p* < 0.05; *n* = 3) (Figure 3E) and a decrease in NO production in the presence of insulin (*p* < 0.05; *n* = 3) (Figure 3F).

These results suggest that alteration of NO production induced by PIN overexpression could be involved in the negative modulation of glucose uptake in L6 myocytes.

### 3.4. Effect of the Pharmacological Inhibitor of nNOS, L-NAME, on Glucose Uptake and GLUT4 Translocation in L6 and Primary Muscle Cells

N-ω-nitro-L-arginine methyl ester (L-NAME), a non-metabolizable analog of arginine, is a competitive inhibitor of NOS activity that blocks catalytic site to reduce the production of NO [25]. We compared the effect of PIN with that of L-NAME, on glucose uptake in L6 and primary myocytes. L-NAME was used at concentrations previously shown to be devoid of nonspecific membrane depolarization effects in pancreatic islets [25]. We first confirmed that its inactive D-analog, D-ω-nitro-L-arginine methyl ester (D-NAME) did not modify glucose uptake in L6 myocytes at the concentrations used (Figure 4A; *n* = 4). Similarly to the effects of PIN overexpression, L-NAME (5 and 10 mM) dose-dependently decreased glucose uptake in the absence of 100 nM insulin by 19 ± 3% (NS) and 30 ± 5% (*p* < 0.01), and more surprisingly in presence of 100 nM insulin by 45 ± 8% (*p* < 0.05) and 71 ± 12% (*p* < 0.001) (*n* = 5) (Figure 4B). In primary myotubes, glucose uptake, unaffected by D-NAME was again dose-dependently reduced by L-NAME (5 and 10 mM), in the absence of insulin (respectively −15.5 ± 4%; *p* < 0.01; −22 ± 2%; and *p* < 0.001) and in the presence of 100 nM insulin (respectively −26 ± 5.5%; *p* < 0.01; −45 ± 3%; and *p* < 0.001) (*n* = 4) (Figure 4C).

Similarly to that observed for PIN overexpression, L-NAME (10 mM) decreased GLUT4 translocation by 22 ± 4% only in the presence of 100 nM insulin in L6 myocytes (*p* < 0.01; *n* = 4) (Figure 4D).

As expected, we confirmed that L-NAME did reduce NO production in the absence (*p* < 0.05) and in the presence of insulin (*p* < 0.001) (*n* = 3), when applied to L6 myocytes (Figure 4E).

In line with the effects of PIN overexpression, our data suggest that an alteration of NO production by a pharmacological inhibitor negatively impacts glucose uptake in L6 and primary muscle cells in basal and insulin-stimulated conditions.

### 3.5. Effect of the NO Donor, SNP, on Glucose Uptake in L6 Muscle Cells

To validate the positive effect of NO on glucose transport, we used the NO donor, sodium nitroprusside (SNP), and found that the compound (from 1 to 6 mM) significantly stimulated glucose uptake in both the presence and the absence of insulin in L6 myocytes (*n* = 5) (Figure 4F).

In agreement with the observed effects of L-NAME, we confirmed to NO is able to enhance glucose uptake in L6 myocytes in basal and insulin-stimulated conditions.

### 3.6. Effects of PIN Silencing on Glucose Uptake in L6 Muscle Cells

In addition to PIN overexpression, we used a selective siRNA to reduce PIN expression in L6 myocytes (−44 ± 5%, *p* < 0.01; *n* = 4) (Figure 5A). Surprisingly, we observed a reduction in glucose uptake by 29 ± 5% (*p* < 0.01) and 62 ± 3% (*p* < 0.001) (*n* = 5) in the absence and presence of 100 nM insulin, respectively (Figure 5B). This data confirms that PIN is an essential protein for glucose transport in muscle cells.

As observed for PIN overexpression, GLUT1 expression was found unchanged (Figure 5C) and GLUT4 slighly enhanced (*p* < 0.01) (Figure 5D), when PIN was silenced in L6 cells. GLUT4 translocation was also reduced in the absence (−17 ± 7%) and in the presence of insulin (−33 ± 5%) (*p* < 0.05; *n* = 4) (Figure 5E). As nNOS was found to modulate the impact of PIN on glucose uptake, we evaluated whether PIN silencing can impact the enzyme. When PIN was downregulated in L6 cells, we observed a decrease in nNOS protein level (*p* < 0.01; *n* = 4) (Figure 5F) whereas NO production is found increased independently of the presence of insulin (*p* < 0.01; *n* = 3) (Figure 5G).

As NO stimulates glucose uptake, this suggests that PIN could also be involved in nNOS-independent effects, as previously shown in pancreatic β-cells for glucose-induced insulin secretion [26].

### 3.7. Design of an Inhibitory Peptide Dissociating PIN Interaction with Its Binding Partners

PIN is able to interact with several protein partners displaying two classes of binding motifs, namely GIQVD and KXTQT [27]. To better understand the role of PIN in glucose uptake and GLUT4 translocation, we designed an inhibitory peptide able to dissociate PIN interaction with its binding partners. For this purpose, we performed a mutational analysis with the binding sequence of nNOS, KDTGIQVDRD (Peptide N), in order to identify a peptide with an increased affinity for PIN; the selected peptide IDVGIQVDWD (Peptide I) displayed three punctual mutations (underlined amino acids). An irrelevant peptide based on another region of nNOS was used as a negative control (peptide C). To validate the specificity of the peptide, we first checked by blast (NCBI Blastp) that the peptide had no homology with mammalian proteins. Using an ELISA format, we showed that the inhibitory peptide was able to dissociate the nNOS-PIN interaction with an IC50 of 0.4 µM, which contrasts with an IC of 300 µM observed for the native peptide (Figure S1A). This data was confirmed by SPR with a maximum binding response of PIN significantly reduced in the presence of 1 µg/mL of inhibitory peptide (300RU versus 60RU), and a total inhibition reached at 50 µg/mL (Figure S1B). Comparatively, the native peptide at 100 µg/mL still gave 40% of the initial response (275 RU versus 110 RU (Figure S1C).

### 3.8. Disruption of PIN with Its Interacting Partners and Consequences on Glucose Uptake in L6 Muscle Cells

The inhibitory peptide was rendered cell-permeant when coupled to Tat-peptide [28]. When the inhibitory peptide was incubated on L6 myocytes, coimmunoprecipitation experiment showed that the direct interaction between PIN and nNOS was found reduced, confirming the in vitro effect of the peptide observed in ELISA and SPR experiments (Figure 5H).

Interestingly, the inhibitory peptide (20 µM) reduced glucose uptake in L6 myocytes by 52 ± 4% (*p* < 0.05) in the absence of and 60 ± 2% in the presence of 100 nM insulin, respectively (*p* < 0.01; *n* = 4) (Figure 5I). We also investigated the effect of the inhibitory peptide in primary myocytes from Zucker *fa/fa* rats, previously shown to remain resistant to insulin in culture [14]. A decreased glucose uptake could be observed in the absence (−17 ± 4%) and presence of insulin (−25 ± 6%) (*p* < 0.05; *n* = 4) (Figure 5J).

This suggests that the ability of PIN to interact with its binding partners is essential for glucose uptake in both physiological and pathological situations.

### 3.9. Expression of Myosin Va and Colocalization with PIN in L6 Muscle Cells

**Figure 6:**
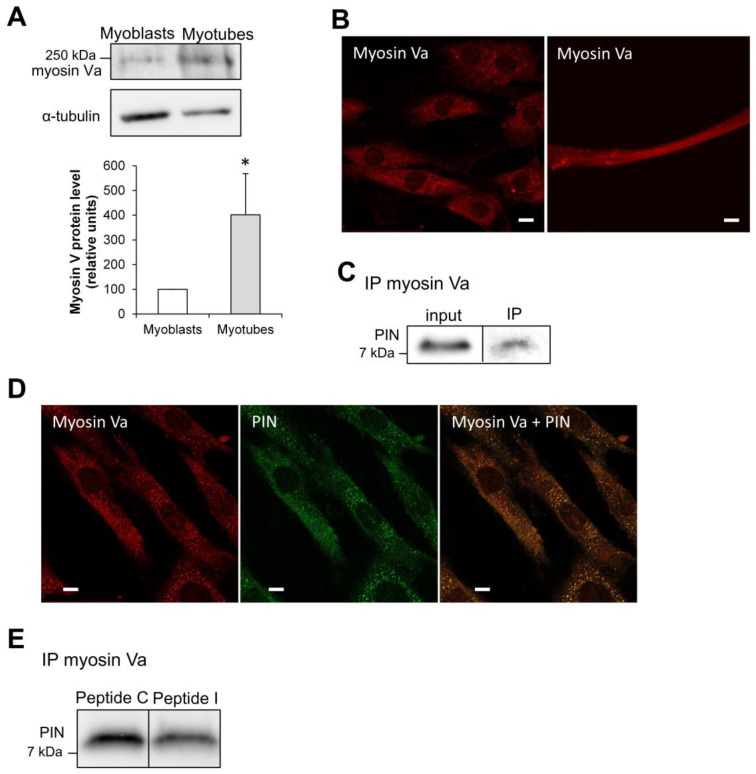
Analysis of myosin Va expression and colocalization with PIN in L6 muscle cells. (**A**) Western blotting analysis of myosin Va expression in L6 myoblasts and L6 myotubes. (**B**) Cellular localization of myosin Va in L6 myocytes (left picture) and L6 myotubes (right picture) by immunofluorescence. (**C**) Coimmunoprecipitation (IP) of myosin Va with PIN in L6 myocytes. (**D**) Colocalization of myosin Va (red) and PIN (green) in L6 myocytes by immunofluorescence. Scale bar is for 10 µm. (**E**) Coimmunoprecipitation of myosin Va with PIN in the presence of the control (Peptide C) versus the inhibitory (Peptide I) peptide in L6 myocytes. * *p* < 0.05.

Among PIN binding partners, myosin V is the sole molecular motor implicated in the traffic of intracellular cargo along actin filaments [2]. L6 myocytes have been shown to express the myosin Va isoform, which is involved in GLUT4 translocation [29]. During differentiation of L6 cells, the protein level of myosin Va strongly increased in myocytes, as compared to myoblasts (Figure 6A). Myosin Va staining appeared punctuated and distributed throughout the cytoplasm of L6 myocytes and also of myotubes (Figure 6B). The direct interaction of myosin Va and PIN has been confirmed by an coimmunoprecipitation experiment performed on cell extracts (Figure 6C). By immunofluorescence staining, we also observed that myosin Va strongly colocalizes with PIN in this cell line (Figure 6D) (Pearson coefficient 0.67 ± 0.048, *n* = 3). This result is consistent with the fact that PIN is a light chain for myosin V [2].

Similarly to its effect on nNOS-PIN interaction, the inhibitory peptide was able to dose-dependently reduce the direct interaction between PIN and myosin Va, using an ELISA format performed with myosin Va purified from rat brain [22] (Figure S1D). When the inhibitory peptide was incubated on L6 myocytes, coimmunoprecipitation experiment showed that direct interaction between PIN and myosin Va was found decreased, as observed in the ELISA format (Figure 6E).

### 3.10. Role of the PIN Binding Partner Myosin Va in Glucose Uptake in L6 Muscle Cells

**Figure 7:**
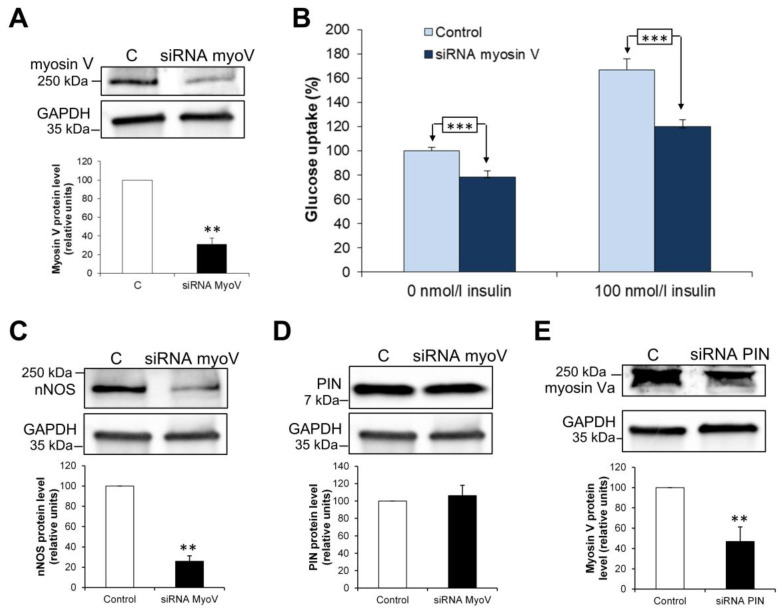
Role of myosin Va in glucose uptake in L6 muscle cells. (**A**) Western blotting analysis of myosin Va under-expression after transfection with myosin Va (siRNA myoV) or control (C) siRNA. (**B**) Effects of myosin Va silencing on glucose uptake in L6 myocytes with or without 100 nM insulin. (**C**) Western blotting analysis of nNOS expression in L6 myocytes after transfection with myosin Va (siRNA myoV) or control (C) siRNA. (**D**) Western blotting analysis of PIN expression in L6 myocytes after transfection with myosin Va (siRNA myoV) or control (C) siRNA. (**E**) Western blotting analysis of myosin Va expression after transfection with PIN siRNA or control (C) siRNA. ** *p* < 0.01 and *** *p* < 0.001.

We used a selective siRNA to silence myosin Va (−69 ± 7%) in L6 myocytes (Figure 7A). The reduced myosin Va expression led to a decrease in glucose uptake reaching 21.5 ± 4.8% (*p* < 0.001) in the absence of and 43 ± 5.8% (*p* < 0.001) (*n* = 6) in the presence of 100 nM insulin (Figure 7B), in line with previously published data on GLUT4 translocation [29]. When myosin V was downregulated in L6 cells, we observed a decrease in nNOS protein level (*p* < 0.01; *n* = 3) (Figure 7C). However, PIN expression remained unchanged (*n* = 5) (Figure 7D), suggesting that it may poorly impact nNOS catalytic activity. Our data thus confirmed that myosin Va is essential for glucose uptake.

Importantly, when PIN was silenced by siRNA, myosin Va expression was subsequently decreased by 53% (Figure 7E). These results suggest that the impairment of glucose uptake found with PIN siRNA is mainly due to myosin Va knockdown and the reduced ability of PIN to interact with binding partners.

### 3.11. Expression of PIN, nNOS and Myosin Va in Skeletal Muscle of Diabetic ZDF Rats

**Figure 8:**
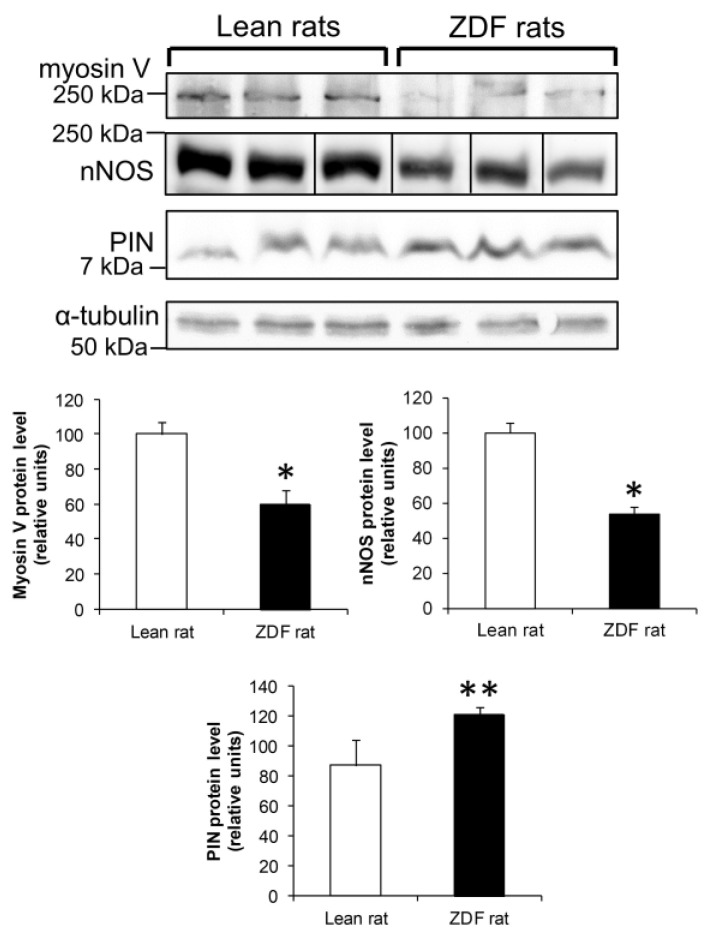
Expression of nNOS, PIN and myosin Va in skeletal muscle from diabetic ZDF rats. Western blotting analysis of nNOS, myosin Va and PIN expression in gastrocnemious muscle from ZDF versus lean rats. * *p* < 0.05 and ** *p* < 0.01.

We finally measured expression of nNOS, PIN and myosinVa in gastrocnemious muscle from 10-week-old ZDF rats. At this age, the animals displayed an elevation of their fasted glycemia reaching 1.28 ± 0.26 g/L, versus 0.79 ± 0.14 g/L for their lean littermates (*p* < 0.01; *n* = 6). As previously observed in muscles form Zucker *fa/fa* rats, nNOS is found down-regulated due to an increased proteosomal degradation of the enzyme in this tissue (*p* < 0.001) [14]. We also observed that PIN expression was found higher (*p* < 0.05), whereas that of myosin Va lower (*p* < 0.05) in ZDF rats than in lean ones (Figure 8).

## 4. Discussion

Our study confirms that PIN is expressed in L6 muscle cells, as previously shown in ventilatory and skeletal muscles [13,30]. Indeed, PIN is a ubiquitous protein, highly expressed in the testis [26], moderately in the brain [31], kidney [32], muscles [13], pancreas [26], and faintly in other tissues. PIN protein level remains unchanged during differentiation of L6 cells into myocytes. In addition, immunofluorescence staining shows that PIN displays two cellular localizations, i.e., the sarcoplasm and the sarcolemma, as previously observed in sections from rodent or human muscles [13,14,30].

Our data demonstrate that PIN overexpression leads to a reduced glucose uptake, under both basal- and insulin-stimulated conditions, in L6 and primary myocytes. Moreover, enhanced PIN protein level induces a decrease in GLUT4 expression and translocation at the plasma membrane. PIN effect on GLUT4 expression probably involves its interaction with a transcription factor able to influence transcriptional activity of GLUT4 promoter. For example, PIN is known to interact with the transcription factor ASCIZ, and high PIN protein levels inhibit its transcriptional activity [33]. ASCIZ and PIN complex is also involved in transcriptional programs leading to B lymphoid cell differentiation [34].

Among PIN partners, nNOS has been described to be involved in skeletal muscles, in the regulation of contractile force, insulin- and exercise-stimulated glucose transport, energy metabolism, and blood flow [15]. PIN has been shown to inhibit NO production [1], although some conflicting data suggest that it binds to nNOS without inhibition [35,36]. In L6 myocytes, we demonstrate that overexpression of PIN decreases nNOS catalytic activity and also its protein level. These results are in line with data obtained in neurons where a reduction in nNOS activity is induced after PIN overexpression [37]. In paraventricular nucleus neurons, angiotensin 2 has also been found to enhance PIN expression, thereby reducing the inhibitory action of NO on neurogenic control of blood pressure [38]. As NO is able to stimulate GLUT4 translocation and/or glucose transport in skeletal muscles in vitro [24,39,40] and in vivo [41], the less release of NO induced by PIN can be responsible for fewer GLUT4 at the plasma membrane and less efficiency of glucose transport. In addition, reduced NO production can also impact GLUT4 mRNA synthesis, as NO has been shown to increase GLUT4 expression through cGMP- and AMPK-dependent mechanism in skeletal muscles [42]. In glomerular podocytes, NO also enhances GLUT4 expression and thereby improves glucose uptake [43].

When compared to the effects of PIN overexpression, the pharmacological inhibitor of nNOS, L-NAME, also displays negative effects on glucose uptake and GLUT4 translocation. Despite the two inhibitors have different mechanisms of action, they are both able to reduce intracellular NO production in L6 cells. Indeed, L-NAME is a non-metabolizable analog of arginine that binds to the active site of NOS enzymes [44], whereas PIN interacts with the NH_2_-terminal part of nNOS to block the enzyme dimerization [1]. From our data obtained with L-NAME and the NO donor SNP, we confirm that (1) NO produced intrinsically by muscle cells is sufficient to exert its metabolic effects, independently of NO produced by eNOS in the vasculature and (2) improvement of glucose uptake by NO clearly occurs in the presence of insulin, and not only in the absence of insulin or after muscle contraction, as previously stated [24,40]. This is supported by the report showing that stimulation of C2C12 cells by insulin induces phosphorylation of nNOS on Ser 1412, thereby increasing NO production [45]. This could explain why the effect of L-NAME is more pronounced in the presence of insulin. Therefore, and in contrast to previous studies [41,46], our data support that NO positively controls glucose transport independently of insulin signaling in both cell line and primary cell models [39,47].

Importantly, nNOS phosphorylation can also depend on cellular redox status as low levels of hydrogen peroxide H_2_O_2_ lead to phosphorylation of nNOS at Ser 1446 and GLUT4 translocation in L6 and C2C12 myotube [48]. Interestingly, in L6 and rat primary myotubes, insulin signaling can also increase intracellular H_2_O_2_ production which promotes glucose uptake [49]. Moreover, NO produced after insulin stimulation and nNOS phosphorylation can S-nitrosylate insulin receptor and IRS1 through SNO-CoA-assisted nitrosylase (SCAN) to exert a negative feedback on insulin signaling [50].

Surprisingly, PIN silencing induced by a specific siRNA, and also decreased glucose uptake and GLUT4 translocation under both basal and insulin-stimulated conditions, as observed for PIN overexpression. This data confirms that PIN is an essential protein for glucose uptake and more broadly for numerous cellular functions, as its total loss-of-function is lethal in fly [12]. Of interest, when PIN is downregulated, we observed an increased nNOS catalytic activity whereas nNOS protein level is found decreased. As opposite to conflicting data suggesting that PIN binds nNOS without inhibition [35,36], this result clearly confirms that depending on the level of PIN, nNOS catalytic activity can be reduced or enhanced in the context of L6 myocytes. PIN silencing also reduced nNOS expression, confirming its essential role in transcriptional programs, through its interaction with transcription factors [7,33]. GLUT4 is also found increased, conforming the positive effect of the NO produced on the transporter expression [42,43].

Overall, our data on glucose uptake suggest that the effect of PIN silencing seems to be poorly dependent on the modulation of nNOS activity, since NO enhances glucose transport. As PIN is now considered as a hub protein [51], this effect is necessary mediated by an interaction with another binding partner. To confirm our hypothesis, we designed an inhibitory peptide based on the binding motif of nNOS to PIN, able to compete with PIN binding partners. This peptide does not display any homology with known mammalian proteins, suggesting it can specifically interact with PIN binding groove. Indeed, PIN monomers form a dimer, thus creating two deep hydrophobic grooves composed of six antiparallel β-strands at the interface of each monomer [52]. In vitro, we found that the inhibitory peptide strongly reduced nNOS-PIN interaction when using recombinant proteins, suggesting that it binds to PIN binding grooves to prevent the binding of other partners. In L6 myocytes, the inhibitory peptide rendered cell-permeant also reduced PIN-nNOS interaction, confirming its ability to interact with PIN binding grooves in a cell context. Interestingly, a strong inhibition of glucose uptake is induced by our peptide, which fully suggests that the mechanisms operating involve a direct interaction of PIN with another binding partner.

Myosin Va is an actin-based cytoskeletal motor involved in cargo transport (for review, see [53]). The molecular motor is a dimeric protein, composed of three distinct parts: (1) the N-terminal head domain containing ATP and actin binding sites; (2) the neck domain displaying 6 IQ motifs bound to calmodulin or calmodulin-like proteins [54]; and (3) the globular tail domain involved in cargo binding and preceded by a coiled coil tail segment. PIN is a light chain of myosin Va, interacting with the tail domain through the non-canonical recognition motif (EDKNTMTD) [27,55]. Exon B, located in the coiled coil domain of myosin Va and present in brain [56], pancreatic β-cells [53], and L6 cells [29] is essential for PIN binding. Biochemical and structural studies revealed that PIN binding to the tail domain of myosin Va allows the folding of this region and promotes the stability of the dimeric protein [55,57], as previously shown for other PIN binding partners [51]. In L6 myocytes, we bring evidence for a direct interaction between PIN and myosin V, as also described in varicosities of nitrergic neurons [58] at postsynaptic sites of neurons [59] and in INS-1 cells [26].

As observed for PIN knockdown, myosin Va silencing reduced glucose uptake under both basal and insulin-stimulated conditions. This effect is ascribed to a reduced GLUT4 translocation at the cell surface of L6-GLUT4*myc* cells [29]. Myosin Va is also involved in GLUT4 translocation in 3T3-L1 adipocytes, after actin phosphorylation by Akt2 upon insulin stimulation [60]. Importantly, PIN knockdown also induced a reduction in myosin Va expression similar to that observed with myosin Va siRNA, confirming its essential role in the observed effects of PIN silencing on glucose uptake. Therefore, the level of myosin Va protein and its ability to bind its partner PIN has a major impact on glucose uptake and GLUT4 translocation.

Finally, in muscle from diabetic ZDF rats, we confirm that nNOS is downregulated as previously observed in muscle from Zucker *fa/fa* rats, due to an increased degradation by the ubiquitin-proteasome system which contributes in part to the reduction in nNOS activity [14]. Our data also show that myosin Va is less expressed, whereas PIN is found increased in muscle from diabetic ZDF rats. Overexpression of PIN can counteract nNOS activity and its positive effect on glucose transport [17]. Myosin V is also reduced, suggesting a reduced ability of the motor to promote GLUT4 translocation [29]. Both mechanisms could participate in the impairment of insulin-mediated glucose transport and utilization observed in type 2 diabetic patients, as a consequence of impaired insulin signaling and GLUT4 translocation (for review, see [61]).

In conclusion, we have identified that PIN modulates glucose uptake and GLUT4 translocation in skeletal muscle through the regulation of NO production and via interactions with the cytoskeletal motor myosin Va. Therefore, pathways involving nNOS and myosin Va could be considered in the future for new therapeutic strategies for insulin resistance in type 2 diabetes.

## Data Availability

The original contributions presented in this study are included in the article/Supplementary Materials. Further inquiries can be directed to the corresponding author(s).

## Supplementary Materials

The following supporting information can be downloaded at https://www.mdpi.com/article/10.3390/antiox15040436/s1, Figure S1: Effects of the inhibitory peptide on nNOS-PIN and nNOS-myosin Va interaction. (A) Effect of increasing concentrations of the inhibitory peptide (peptide I), the native one (peptide N), and an irrelevant peptide (Peptide C) on nNOS-PIN interaction in an ELISA assay. (B) Real-time analysis of nNOS-PIN interaction by SPR in the presence or not of increasing concentrations of the inhibitory peptide (1, 1 µg/mL; 2, 2 µg/mL; 3, 3 µg/mL; 4, 5 µg/ml; 5, 10 µg/mL). (C) Real-time analysis of nNOS-PIN interaction by SPR in the presence of increasing concentrations of the native peptide (1, 20 µg/mL; 2, 50 µg/mL; 3, 100 µg/mL). (D) Effect of increasing concentrations of the inhibitory peptide (peptide I) on myosin Va-PIN interaction in an ELISA assay.

## Abbreviations

eNOS	endothelial nitric oxide synthase
D-NAME	D-ω-nitro-L-arginine methyl ester
L-NAME	N-ω-nitro-L-arginine methyl ester
nNOS	neuronal nitric oxide synthase
NO	nitric oxide
NOS	nitric oxide synthase
PIN	protein inhibitor of neuronal NOS
SNP	sodium nitroprusside
SPR	surface plasmon resonance
ZDF	Zucker Diabetic Fatty

## References

[1] Jaffrey S.R., Snyder S.H. (1996). PIN: An associated protein inhibitor of neuronal nitric oxide synthase. Science.

[2] Benashski S.E., Harrison A., Patel-King R.S., King S.M. (1997). Dimerization of the highly conserved light chain shared by dynein and myosin V. J. Biol. Chem..

[3] Puthalakath H., Huang D.C., O_Reilly L.A., King S.M., Strasser A. (1999). The proapoptotic activity of the Bcl-2 family member Bim is regulated by interaction with the dynein motor complex. Mol. Cell.

[4] Haraguchi K., Satoh K., Yanai H., Hamada F., Kawabuchi M., Akiyama T. (2000). The hDLG-associated protein DAP interacts with dynein light chain and neuronal nitric oxide synthase. Genes Cells.

[5] Schnorrer F., Bohmann K., Nusslein-Volhard C. (2000). The molecular motor dynein is involved in targeting swallow and bicoid RNA to the anterior pole of Drosophila oocytes. Nat. Cell Biol..

[6] Herzig R.P., Andersson U., Scarpulla R.C. (2000). Dynein light chain interacts with NRF-1 and EWG, structurally and functionally related transcription factors from humans and drosophila. J. Cell Sci..

[7] Kirkham J.K., Park S.H., Nguyen T.N., Lee J.H., Gunzl A. (2015). Dynein Light Chain LC8 Is Required for RNA Polymerase I-Mediated Transcription in Trypanosoma brucei, Facilitating Assembly and Promoter Binding of Class I Transcription Factor A. Mol. Cell Biol..

[8] Raux H., Flamand A., Blondel D. (2000). Interaction of the rabies virus P protein with the LC8 dynein light chain. J. Virol..

[9] Lightcap C.M., Kari G., Arias-Romero L.E., Chernoff J., Rodeck U., Williams J.C. (2009). Interaction with LC8 is required for Pak1 nuclear import and is indispensable for zebrafish development. PLoS ONE.

[10] Jin M., Yamada M., Arai Y., Nagai T., Hirotsune S. (2014). Arl3 and LC8 regulate dissociation of dynactin from dynein. Nat. Commun..

[11] Wong D.M., Li L., Jurado S., King A., Bamford R., Wall M., Walia M.K., Kelly G.L., Walkley C.R., Tarlinton D.M. (2016). The Transcription Factor ASCIZ and Its Target DYNLL1 Are Essential for the Development and Expansion of MYC-Driven B Cell Lymphoma. Cell Rep..

[12] Dick T., Ray K., Salz H.K., Chia W. (1996). Cytoplasmic dynein (ddlc1) mutations cause morphogenetic defects and apoptotic cell death in Drosophila melanogaster. Mol. Cell Biol..

[13] Guo Y., Petrof B.J., Hussain S.N. (2001). Expression and localization of protein inhibitor of neuronal nitric oxide synthase in Duchenne muscular dystrophy. Muscle Nerve.

[14] Mezghenna K., Leroy J., Azay-Milhau J., Tousch D., Castex F., Gervais S., Delgado-Betancourt V., Gross R., Lajoix A.D. (2014). Counteracting neuronal nitric oxide synthase proteasomal degradation improves glucose transport in insulin-resistant skeletal muscle from Zucker fa/fa rats. Diabetologia.

[15] DeFronzo R.A., Jacot E., Jequier E., Maeder E., Wahren J., Felber J.P. (1981). The effect of insulin on the disposal of intravenous glucose. Results from indirect calorimetry and hepatic and femoral venous catheterization. Diabetes.

[16] Vargas E., Podder V., Sepulveda M.A.C. (2023). Physiology, Glucose Transporter Type 4. StatPearls [Internet].

[17] McConell G.K., Rattigan S., Lee-Young R.S., Wadley G.D., Merry T.L. (2012). Skeletal muscle nitric oxide signaling and exercise: A focus on glucose metabolism. Am. J. Physiol. Endocrinol. Metab..

[18] Cui Z., Chen X., Lu B., Park S.K., Xu T., Xie Z., Xue P., Hou J., Hang H., Yates J.R. (2009). Preliminary quantitative profile of differential protein expression between rat L6 myoblasts and myotubes by stable isotope labeling with amino acids in cell culture. Proteomics.

[19] Bolte S., Cordelieres F.P. (2006). A guided tour into subcellular colocalization analysis in light microscopy. J. Microsc..

[20] Costes S.V., Daelemans D., Cho E.H., Dobbin Z., Pavlakis G., Lockett S. (2004). Automatic and quantitative measurement of protein-protein colocalization in live cells. Biophys. J..

[21] Henstridge D.C., Drew B.G., Formosa M.F., Natoli A.K., Cameron-Smith D., Duffy S.J., Kingwell B.A. (2009). The effect of the nitric oxide donor sodium nitroprusside on glucose uptake in human primary skeletal muscle cells. Nitric Oxide.

[22] Melo H.C., Coelho M.V. (2007). A new method to precipitate myosin V from rat brain soluble fraction. Acta Biochim. Pol..

[23] Rudich A., Konrad D., Török D., Ben-Romano R., Huang C., Niu W., Garg R.R., Wijesekara N., Germinario R.J., Bilan P.J. (2003). Indinavir uncovers different contributions of GLUT4 and GLUT1 towards glucose uptake in muscle and fat cells and tissues. Diabetologia.

[24] Young M.E., Radda G.K., Leighton B. (1997). Nitric oxide stimulates glucose transport and metabolism in rat skeletal muscle in vitro. Biochem. J..

[25] Mezghenna K., Pomies P., Chalancon A., Castex F., Leroy J., Niclauss N., Nadal B., Cambier L., Cazevieille C., Petit P. (2011). Increased neuronal nitric oxide synthase dimerisation is involved in rat and human pancreatic beta cell hyperactivity in obesity. Diabetologia.

[26] Lajoix A.D., Badiou S., Peraldi-Roux S., Chardes T., Dietz S., Aknin C., Tribillac F., Petit P., Gross R. (2006). Protein inhibitor of neuronal nitric oxide synthase (PIN) is a new regulator of glucose-induced insulin secretion. Diabetes.

[27] Lajoix A.D., Gross R., Aknin C., Dietz S., Granier C., Laune D. (2004). Cellulose membrane supported peptide arrays for deciphering protein-protein interaction sites: The case of PIN, a protein with multiple natural partners. Mol. Divers..

[28] Patel S.G., Sayers E.J., He L., Narayan R., Williams T.L., Mills E.M., Allemann R.K., Luk L.Y.P., Jones A.T., Tsai Y.H. (2019). Cell-penetrating peptide sequence and modification dependent uptake and subcellular distribution of green florescent protein in different cell lines. Sci. Rep..

[29] Sun Y., Chiu T.T., Foley K.P., Bilan P.J., Klip A. (2014). Myosin Va mediates Rab8A-regulated GLUT4 vesicle exocytosis in insulin-stimulated muscle cells. Mol. Biol. Cell.

[30] Guo Y., Greenwood M.T., Petrof B.J., Hussain S.N. (1999). Expression and regulation of protein inhibitor of neuronal nitric oxide synthase in ventilatory muscles. Am. J. Respir. Cell Mol. Biol..

[31] Greenwood M.T., Guo Y., Kumar U., Beausejours S., Hussain S.N. (1997). Distribution of protein inhibitor of neuronal nitric oxide synthase in rat brain. Biochem. Biophys. Res. Commun..

[32] Roczniak A., Levine D.Z., Burns K.D. (2000). Localization of protein inhibitor of neuronal nitric oxide synthase in rat kidney. Am. J. Physiol. Renal Physiol..

[33] Jurado S., Conlan L.A., Baker E.K., Ng J.L., Tenis N., Hoch N.C., Gleeson K., Smeets M., Izon D., Heierhorst J. (2012). ATM substrate Chk2-interacting Zn2+ finger (ASCIZ) Is a bi-functional transcriptional activator and feedback sensor in the regulation of dynein light chain (DYNLL1) expression. J. Biol. Chem..

[34] King A., Li L., Wong D.M., Liu R., Bamford R., Strasser A., Tarlinton D.M., Heierhorst J. (2017). Dynein light chain regulates adaptive and innate B cell development by distinctive genetic mechanisms. PLoS Genet..

[35] Rodriguez-Crespo I., Straub W., Gavilanes F., de Montellano P.R.O. (1998). Binding of dynein light chain (PIN) to neuronal nitric oxide synthase in the absence of inhibition. Arch. Biochem. Biophys..

[36] Parhad S.S., Jaiswal D., Ray K., Mazumdar S. (2016). The protein inhibitor of nNOS (PIN/DLC1/LC8) binding does not inhibit the NADPH-dependent heme reduction in nNOS, a key step in NO synthesis. Biochem. Biophys. Res. Commun..

[37] Yang J., Dennison N.N., Reiss C.S. (2008). PIN: A novel protein involved in IFN-gamma accumulation of NOS-1 in neurons. DNA Cell Biol..

[38] Sharma N.M., Haibara A.S., Katsurada K., Liu X., Patel K.P. (2020). Central angiotensin II-Protein inhibitor of neuronal nitric oxide synthase (PIN) axis contribute to neurogenic hypertension. Nitric Oxide.

[39] Etgen G.J., Fryburg D.A., Gibbs E.M. (1997). Nitric oxide stimulates skeletal muscle glucose transport through a calcium/contraction- and phosphatidylinositol-3-kinase-independent pathway. Diabetes.

[40] Balon T.W., Nadler J.L. (1997). Evidence that nitric oxide increases glucose transport in skeletal muscle. J. Appl. Physiol..

[41] Roy D., Perreault M., Marette A. (1998). Insulin stimulation of glucose uptake in skeletal muscles and adipose tissues in vivo is NO dependent. Am. J. Physiol..

[42] Lira V.A., Soltow Q.A., Long J.H., Betters J.L., Sellman J.E., Criswell D.S. (2007). Nitric oxide increases GLUT4 expression and regulates AMPK signaling in skeletal muscle. Am. J. Physiol. Endocrinol. Metab..

[43] Rogacka D., Audzeyenka I., Rachubik P., Szrejder M., Typiak M., Angielski S., Piwkowska A. (2021). Involvement of nitric oxide synthase/nitric oxide pathway in the regulation of SIRT1-AMPK crosstalk in podocytes: Impact on glucose uptake. Arch. Biochem. Biophys..

[44] Klatt P., Schmidt K., Brunner F., Mayer B. (1994). Inhibitors of brain nitric oxide synthase. Binding kinetics, metabolism, and enzyme inactivation. J. Biol. Chem..

[45] Hinchee-Rodriguez K., Garg N., Venkatakrishnan P., Roman M.G., Adamo M.L., Masters B.S., Roman L.J. (2013). Neuronal nitric oxide synthase is phosphorylated in response to insulin stimulation in skeletal muscle. Biochem. Biophys. Res. Commun..

[46] Kapur S., Bedard S., Marcotte B., Cote C.H., Marette A. (1997). Expression of nitric oxide synthase in skeletal muscle: A novel role for nitric oxide as a modulator of insulin action. Diabetes.

[47] Higaki Y., Hirshman M.F., Fujii N., Goodyear L.J. (2001). Nitric oxide increases glucose uptake through a mechanism that is distinct from the insulin and contraction pathways in rat skeletal muscle. Diabetes.

[48] Kellogg D.L., McCammon K.M., Hinchee-Rodriguez K.S., Adamo M.L., Roman L.J. (2017). Neuronal nitric oxide synthase mediates insulin- and oxidative stress-induced glucose uptake in skeletal muscle myotubes. Free Radic. Biol. Med..

[49] Contreras-Ferrat A., Llanos P., Vásquez C., Espinosa A., Osorio-Fuentealba C., Arias-Calderon M., Lavandero S., Klip A., Hidalgo C., Jaimovich E. (2014). Insulin elicits a ROS-activated and an IP_3_-dependent Ca^2+^ release, which both impinge on GLUT4 translocation. J. Cell Sci..

[50] Zhou H.L., Grimmett Z.W., Venetos N.M., Stomberski C.T., Qian Z., McLaughlin P.J., Bansal P.K., Zhang R., Reynolds J.D., Premont R.T. (2023). An enzyme that selectively S-nitrosylates proteins to regulate insulin signaling. Cell.

[51] Barbar E. (2008). Dynein light chain LC8 is a dimerization hub essential in diverse protein networks. Biochemistry.

[52] Liang J., Jaffrey S.R., Guo W., Snyder S.H., Clardy J. (1999). Structure of the PIN/LC8 dimer with a bound peptide. Nat. Struct. Biol..

[53] Li J., Lu Q., Zhang M. (2016). Structural Basis of Cargo Recognition by Unconventional Myosins in Cellular Trafficking. Traffic.

[54] Espindola F.S., Suter D.M., Partata L.B., Cao T., Wolenski J.S., Cheney R.E., King S.M., Mooseker M.S. (2000). The light chain composition of chicken brain myosin-Va: Calmodulin, myosin-II essential light chains, and 8-kDa dynein light chain/PIN. Cell Motil. Cytoskeleton.

[55] Bodor A., Radnai L., Hetenyi C., Rapali P., Lang A., Kover K.E., Perczel A., Wahlgren W.Y., Katona G., Nyitray L. (2014). DYNLL2 dynein light chain binds to an extended linear motif of myosin 5a tail that has structural plasticity. Biochemistry.

[56] Varadi A., Tsuboi T., Rutter G.A. (2005). Myosin Va transports dense core secretory vesicles in pancreatic MIN6 beta-cells. Mol. Biol. Cell.

[57] Wagner W., Fodor E., Ginsburg A., Hammer J.A. (2006). The binding of DYNLL2 to myosin Va requires alternatively spliced exon B and stabilizes a portion of the myosin’s coiled-coil domain. Biochemistry.

[58] Chaudhury A., He X.D., Goyal R.K. (2011). Myosin Va plays a key role in nitrergic neurotransmission by transporting nNOSalpha to enteric varicosity membrane. Am. J. Physiol. Gastrointest. Liver Physiol..

[59] Naisbitt S., Valtschanoff J., Allison D.W., Sala C., Kim E., Craig A.M., Weinberg R.J., Sheng M. (2000). Interaction of the postsynaptic density-95/guanylate kinase domain-associated protein complex with a light chain of myosin-V and dynein. J. Neurosci..

[60] Yoshizaki T., Imamura T., Babendure J.L., Lu J.C., Sonoda N., Olefsky J.M. (2007). Myosin 5a is an insulin-stimulated Akt2 (protein kinase Bbeta) substrate modulating GLUT4 vesicle translocation. Mol. Cell Biol..

[61] Sylow L., Tokarz V.L., Richter E.A., Klip A.A. (2021). The many actions of insulin in skeletal muscle, the paramount tissue determining glycemia. Cell Metab..

