# Supplementary material for: PIN (Protein Inhibitor of Neuronal Nitric Oxide Synthase) Modulates Glucose Uptake Through NO-Dependent and Independent Mechanisms in Rat Muscle Cells
Source: Antioxidants (Basel). 2026 Mar 31;15(4):436. doi: 10.3390/antiox15040436 (PMC13114162; doi:10.3390/antiox15040436)
Supplement: Supplementary file 1 [file antioxidants-15-00436-s001.zip › antioxidants-4202300-supplementary.pdf]

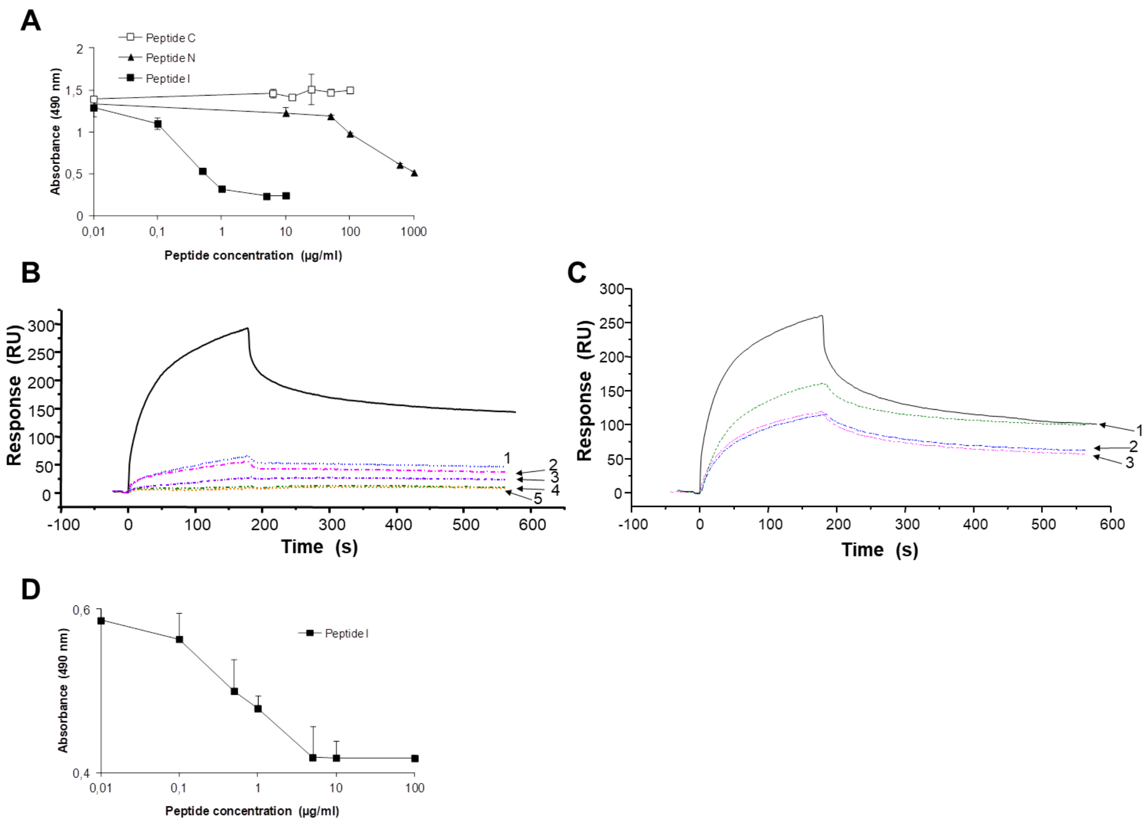

Figure S1. Effects of the inhibitory peptide on nNOS-PIN and nNOS-myosin Va interaction. (A) Effect of increasing concentrations of the inhibitory peptide (peptide I), the native one (peptide N), and an irrelevant peptide (Peptide C) on nNOS-PIN interaction in an ELISA assay. (B) Real-time analysis of nNOS-PIN interaction by SPR in the presence or not of increasing concentrations of the inhibitory peptide (1, 1µg/ml; 2, 2µg/ml; 3, 3µg/ml; 4, 5 µg/ml; 5, 10µg/ml). (C) Real-time analysis of nNOS-PIN interaction by SPR in the presence of increasing concentrations of the native peptide (1, 20µg/ml; 2, 50µg/ml; 3, 100µg/ml). (D) Effect of increasing concentrations of the inhibitory peptide (peptide I) on myosin Va-PIN interaction in an ELISA assay.
